# Supplementary material for: Causal Relationship between Aging and Anorexia Nervosa: A White-Matter-Microstructure-Mediated Mendelian Randomization Analysis
Source: Biomedicines. 2024 Aug 16;12(8):1874. doi: 10.3390/biomedicines12081874 (PMC11351342; doi:10.3390/biomedicines12081874)
Supplement: Supplementary file 1 [file biomedicines-12-01874-s001.zip › Supplementary Figure/Figure S4/Singe SNP plot of MR analysis for TL to ubm-b-1872.pdf]

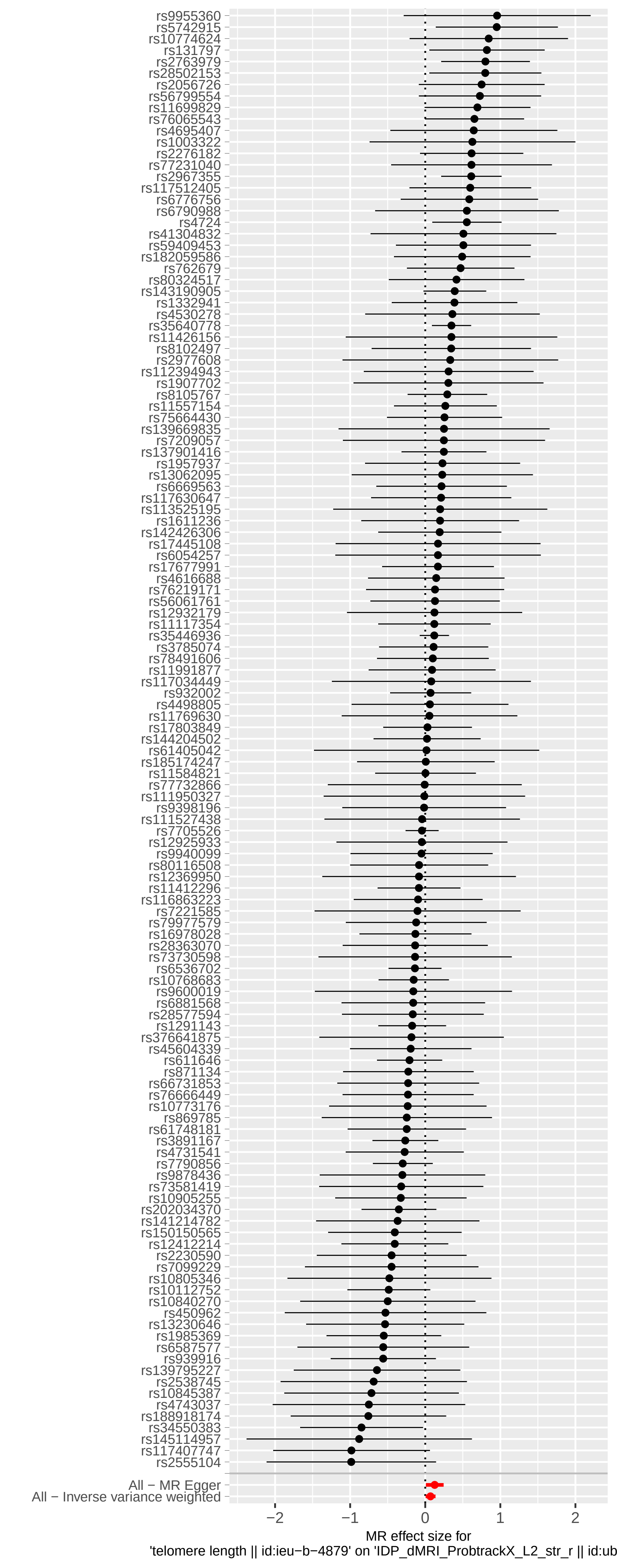

rs9955360
rs5742915
rs10774624
rs131797
rs2763979
rs28502153
rs2056726
rs56799554
rs11699829
rs76065543
rs4695407
rs1003322
rs2276182
rs77231040
rs2967355
rs117512405
rs6776756
rs6790988
rs4724
rs41304832
rs59409453
rs182059586
rs762679
rs80324517
rs143190905
rs1332941
rs4530278
rs35640778
rs11426156
rs8102497
rs2977608
rs112394943
rs1907702
rs8105767
rs11557154
rs75664430
rs139669835
rs7209057
rs137901416
rs1957937
rs13062095
rs6669563
rs117630647
rs113525195
rs1611236
rs142426306
rs17445108
rs6054257
rs17677991
rs4616688
rs76219171
rs56061761
rs12932179
rs11117354
rs35446936
rs3785074
rs78491606
rs11991877
rs117034449
rs932002
rs4498805
rs11769630
rs17803849
rs144204502
rs61405042
rs185174247
rs11584821
rs77732866
rs111950327
rs9398196
rs111527438
rs7705526
rs12925933
rs9940099
rs80116508
rs12369950
rs11412296
rs116863223
rs7221585
rs79977579
rs16978028
rs28363070
rs73730598
rs6536702
rs10768683
rs9600019
rs6881568
rs28577594
rs1291143
rs376641875
rs45604339
rs611646
rs871134
rs66731853
rs76666449
rs10773176
rs869785
rs61748181
rs3891167
rs4731541
rs7790856
rs9878436
rs73581419
rs10905255
rs202034370
rs141214782
rs150150565
rs12412214
rs2230590
rs7099229
rs10805346
rs10112752
rs10840270
rs450962
rs13230646
rs1985369
rs6587577
rs939916
rs139795227
rs2538745
rs10845387
rs4743037
rs188918174
rs34550383
rs145114957
rs117407747
rs2555104
All − MR Egger
All − Inverse variance weighted
−2
−1
0
1
2
MR effect size for
'telomere length || id:ieu−b−4879' on 'IDP_dMRI_ProbtrackX_L2_str_r || id:ub
